# KC21 Peptide Inhibits Angiogenesis and Attenuates Hypoxia-Induced Retinopathy

**DOI:** 10.1007/s12265-019-09865-6

**Published:** 2019-02-21

**Authors:** Chi-Sheng Lu, Yi-Nan Lee, Shin-Wei Wang, Yih-Jer Wu, Cheng-Huang Su, Chin-Ling Hsieh, Ting Yi Tien, Bo-Jeng Wang, Min-Che Chen, Chun-Wei Chen, Hung-I Yeh

**Affiliations:** 1 Present Address: Departments of Medical Research, Mackay Memorial Hospital, Taipei, 10449 Taiwan; 2 Virginia Contract Research Organization Co., Ltd, Taipei, 11491 Taiwan; 3 Department of Medicine, Mackay Medical College, New Taipei City, 25245 Taiwan; 4 Asclepiumm Taiwan Co., Ltd, New Taipei City, 25160 Taiwan; 5 Departments of Medical Research and Internal Medicine, Mackay Memorial Hospital, No. 92, Sec. 2, Zhongshan N. Rd, Taipei City, 10449 Taiwan

**Keywords:** Desmoglein-2, Therapeutic peptide, Angiogenesis, Neovascularization, Endothelial colony-forming cells

## Abstract

Desmogleins (Dsg2) are the major components of desmosomes. Dsg2 has five extracellular tandem cadherin domains (EC1-EC5) for cell-cell interaction. We had previously confirmed the Dsg2 antibody and its epitope (named KC21) derived from EC2 domain suppressing epithelial-mesenchymal transition and invasion in human cancer cell lines. Here, we screened six peptide fragments derived from EC2 domain and found that KR20, the parental peptide of KC21, was the most potent one on suppressing endothelial colony-forming cell (ECFC) tube-like structure formation. KC21 peptide also attenuated migration but did not disrupt viability and proliferation of ECFCs, consistent with the function to inhibit VEGF-mediated activation of p38 MAPK but not AKT and ERK. Animal studies showed that KC21 peptides suppressed capillary growth in Matrigel implant assay and inhibited oxygen-induced retinal neovascularization. The effects were comparable to bevacizumab (Bev). In conclusion, KC21 peptide is an angiogenic inhibitor potentially useful for treating angiogenesis-related diseases.

**Electronic supplementary material:**

The online version of this article (10.1007/s12265-019-09865-6) contains supplementary material, which is available to authorized users.

## Introduction

Desmosomes provide strong adhesion to maintain tissue function and organ architecture. Organs that frequently experience mechanical stress, such as the skin and heart, particularly express abundant desmosomes to provide plasma membrane attachment sites for adjacent cells [1]. Desmosomes are adhesive intercellular junctions comprising two cadherin proteins, desmogleins (Dsg) and desmocollins [2]. Human genome encodes four desmogleins (Dsg1–4) which are single-pass transmembrane proteins with five extracellularly tandem conserved cadherin domains (EC1-EC5) and an intracellular domain that bind to intermediate filaments via adaptor proteins, desmoplakin and plakoglobin [1]. Intercellular junctions of cadherin binding sites are composed of EC1 domains revealed by electron tomography studies of native desmosomes [3, 4]. The specificity of adhesion had been confirmed by function-blocking peptides derived from EC1 domain [5]. Differentially proteolytic cleavage fragments containing EC domains had been determined in human cancer lines [6]. Clinically, shedding of Dsg2 extracellular domains are detected in patients with ulcerative colitis [7]. Mutations of Dsg2 are detected in patients with arrhythmogenic right ventricular cardiomyopathy (ARVC) [8], and expression of Dsg2 is increased in several epithelial-derived malignancies including basal-cell carcinomas, squamous cell carcinomas, and metastatic prostate cancer [9–11]. These studies show the importance of Dsg2 homeostasis for the regulation of signaling in cell proliferation, migration, and epithelial-mesenchymal transition (EMT).

The therapeutic potential of endothelial progenitor cells (EPCs) has gained great interest since the observations that a significant number decrease of circulating EPCs was detected in patients with severe conditions, such as diabetes and repeated hospitalization for heart attacks [12]. EPCs isolated from peripheral bloods consistently produce two distant subtypes which had been named as early EPCs and endothelial colony-forming cells (ECFCs), also called late EPCs for their late appearance in culture. Early EPCs, which produce paracrine factors, have limited culturing passages, and ECFCs, which directly incorporate into vasculature, have a strong growth capacity. Intramuscular injection of human ECFCs rescues blood perfusion of hindlimb ischemic mice [13] that provides rationale for clinical trials using ECFC infusion as ischemic cardiovascular disease therapy [14].

Previously, we had identified the antagonist role of Dsg2 on cancer metastasis [15]. Polyclonal Dsg2 antibody and the immunogenic epitope derived from EC2 domain suppress EMT and invasion of human melanoma, breast cancer, and prostate cancer cells, consistent with the observation that Dsg2 exhibits a non-adhesive function for cell migration and morphogenesis [1, 5, 6]. Here, we use Dsg2 antibody and its immunogeic peptide KC21 to test their effects on the control of vessel overgrowth in vivo and to screen the candidates involved in Dsg2-mediated ECFC angiogenesis.

## Methods

### Isolation, Characterization, and Culture of Human ECFCs

Ethical approval (No. 15MMHIS112) was granted by the Mackay Memorial Hospital Institutional Review Board, Taipei, Taiwan. Informed consent was obtained from healthy donors before the collection of peripheral blood (80 mL). The peripheral blood mononuclear cells (PBMCs) obtained from healthy donors were fractionated from other blood components by centrifugation. EPCs were isolated using CD34 MicroBead kit and MACS cell separation system (Miltenyi Biotec). In this study, PBMCs were cultured for 28 days to get ECFCs (late ECFCs) as described [16]. ECFCs were defined as CD34^+^KDR^+^AC133^+^CD31^+^ as described [13]. ECFCs were cultured in MV2 complete medium (PromoCell, Germany) with hEGF (5 ng/ml), hVEGF (0.5 ng/ml), hFGF-B (10 ng/ml), IGF-1(20 ng/ml), ascorbic acid (1 μg/ml), hydrocortisone (0.2 μg/ml), and 20% fetal bovine serum. 1 × 10^4^ cells/cm^2^ were seeded on 1% gelatin-coated dish (BD Biosciences) and maintained in the 37 °C incubator under a humidified 95% air and 5% CO_2_ atmosphere.

### Cell Viability and Proliferation Analysis

Cell viability was measured using the cell counting kit-8 (CCK-8) (Sigma-Aldrich) to reflect the dehydrogenase activity of living cells. ECFCs were seeded onto 96-well plates and treated with Dsg2-derived peptides (100, 200, and 400 μM). Twenty-four hours later, CCK-8 solutions were added to each well for 4 h, and the medium was harvested for the measurement of absorbance at 450 nm using a microplate reader. For cell proliferation assay, ECFCs were treated with Dsg2-derived peptide (100, 200, and 400 μM) for 4 h and then fixed. Cells labeled with 5-bromo-2′-deoxyuridine (BrdU) were subsequently identified with a primary antibody against BrdU and visualized with a secondary antibody conjugated with horseradish peroxidase using tetramethylbenzidine as a substrate.

### Immunohistochemistry

Immunostaining of cell cultures was described previously [13]. Antibodies used and dilution ratio were rabbit antiDsg2 (1:100, [EPR6767(B)], GeneTex), mouse antiplakoglobin (1: 200, clone 15/γ-Catenin, BD Biosciences), and mouse antiPECAM1 (1: 100, clone JC70A, Dako). Rat monoclonal antiendomucin (1: 100, clone V.7C7.1, abcam). Cells were fixed with −20 °C methanol for 10 min. Phalloidin (1: 1000, PHDG1, cytoskeleton) staining was performed at the step of secondary antibody incubation for 2 h at room temperature. Fluorescent images were acquired by confocal microscope (TCS SP5, Leica).

### Zymography Assay

ECFCs were seeded at 80% confluence on 60-mm dishes in MV2 complete medium. Next day, cells were incubated in MV2 medium with 2% FBS with various concentrations of KC21 peptides for 24 h. Fifty μg of the conditioned medium was analyzed by 10% zymogram gel containing 0.1% gelatin [17]. After electrophoresis, the gels were washed and incubated at 37 °C for 24 h. The gels were stained with Coomassie Blue to visualize proteinase activity. The digested area appeared clear over a blue background, indicating the location of matrix metalloproteinase 2 and 9 (MMP2 and 9) activity. MMP3 activity was determined by casein zymography [18].

### PAI-1 Activity Assay

ECFCs were treated with KC21 or scramble peptides for 24 h. Conditioned media were harvested for PAI-1 activity determination. PAI activity assay kit (Chemicon) utilized a chromogenic substrate cleaved by active uPA and detected by its optical density at 405 nm. Addition of PAI-1 in conditioned media blocked the cleavage of substrate by uPA. The relative PAI-1 activity was obtained by plotting with the standard curve of 10 units of uPA inhibited by a series dilution of PAI-1 incubated at 37 °C for 2 h as the assay instructions described.

### Western Blot

ECFCs were lysed with SB-20 buffer (0.2 g/mL SDS, 10 mM EDTA, 100 mM Tris-HCl, pH 6.8), and protein concentrations were determined by modified Lowry’s method. Aliquots of cell lysates were loaded into 10% SDS-polyacrylamide gels, electrophoresed, and transblotted onto polyvinylidene fluoride membranes (Millipore). The blots were blocked with 10% bovine serum albumin for 1 h and probed with indicated primary antibody for two hours. The blots were further incubated with alkaline phosphatase-conjugated secondary antibodies for 1 hour at a room temperature. Immunoreactivity was visualized using CDP-star system (Roche) according to the manufacturer’s instruction. Primary antibody for Dsg2 (Santa Cruz), p38, p-p38, Akt, p-Akt, ERK, p-ERK, MMP9, and PAI-1 (Cell signaling) were diluted with PBS in 1 to 1000.

### Wound Healing Assay

ECFCs were grown on twenty-four-well plates to reach confluence. Cell-free gap was generated using SPLScar™ Block (0.5 mm width, #201905, SPL Life Sciences, Korea) and photographed by optical microscopy (Leica, Germany) at × 40 magnification as basal line. After 4-h culture, cells were fixed and imagined to measure new growth areas using Image-J software (NIH). The ratio of the new migration area was calculated relative to the initial wound area and normalized to that for PBS-treated cells as described [19].

### Matrigel Tube Formation Assay and Quantification

Growth factor-reduced Matrigel (BD Biosciences) was thawed at 4 °C before use. Twenty-four-well plates were coated with Matrigel (200 μL/well) and polymerized for 30 min at 37 °C. ECFCs resuspended with various concentrations of KC21 peptides, scramble peptides, and antiDsg2 antibody (10 ng/ml or bev (0.25 μg/mL)) were seeded on Matrigel-coated wells at a density of 5 × 10^4^ cells in MV2 medium containing 2% FBS for 24 h at 37 °C in a 5% CO_2_ humidified incubator. Each sample was tested in triplicate on the same plate, and wells were photographed with a Leica microscope with camera (× 40 magnification). Five fields were randomly chosen in each well to measure tube length and junction number manually using Image-Pro Plus 6.0 (Rockville, MD). Total tube length and junction number per field were calculated.

### Animal Experiments

All animal experiments were approved by the Institutional Animal Care and Use Committee of the Mackay Memorial Hospital. C57BL/6 mice were kept and bred in accordance with the institutional ethical committee guidance (approval number: MMH-A-S-105-67).

### Matrigel Plug Assay

To assess the antiangiogenic effects of KC21 peptides in vivo, growth factor-reduced liquid Matrigel (0.5 mL) containing heparin (60 U/mL), VEGF (10 ng/mL, with the exception of control), and KC21 peptides or scramble peptides were subcutaneously injected into the mice near the abdominal midline. Seven days after injection, mice were euthanized and Matrigel plugs were surgically removed. For macroscopic analysis of angiogenesis, hemoglobin content in Matrigel was measured with Drabkin’s reagent kit 525 (Sigma-Aldrich).

### Oxygen-Induced Retinopathy Assay

Retinal neovascularization was induced by the use of a well-established murine model of oxygen-induced retinopathy [20]. Neonatal mouse (C57BL/6) pups at postnatal day 7 (P7) with their nursing mothers were maintained for 5 days in 75% oxygen and then returned to room air (relative hypoxia) to produce retinal neovascularization at P12. PBS, scramble, KC21 peptides (25 μg), or Bev (10 µg) were then administered by intravitreal injection into mouse eyes at P12. The animals were sacrificed and the mouse eyes were enucleated at P17. Mouse eye cups were fixed in 4% paraformaldehyde for 2 h. The retinas were carefully separated from eye cups and then incubated with fluorescein-labeled isolectin-B4 (Life technologies) at 4 °C overnight. Samples were mounted with Vectashield medium (Vector Laboratories), and the isolectin labeling was examined by using the × 20 objective of a Leica TCS SP5 confocal microscope. Fluorescence volume measurements were recorded by creating image stacks of optical slices within lesions with QWIN software.

## Results

### Characterization of Peptides Derived from EC2 Domain of Dsg2 for Suppressing ECFC Tube Formation

We had identified the antagonist role of EC2 domain in suppressing EMT and invasion of human cancer cells [15] . In this study, we used ECFCs to test the effects of Dsg2-derived peptides on angiogenesis. At first, human EPCs were harvested from the peripheral blood mononuclear cells (PBMCs) of healthy donors and characterized with CD34^+^KDR^+^AC133^+^CD31^+^ [21]. The fractions of defined ECFCs were determined by flow cytometry (Fig. S1).

**Fig. 1:**
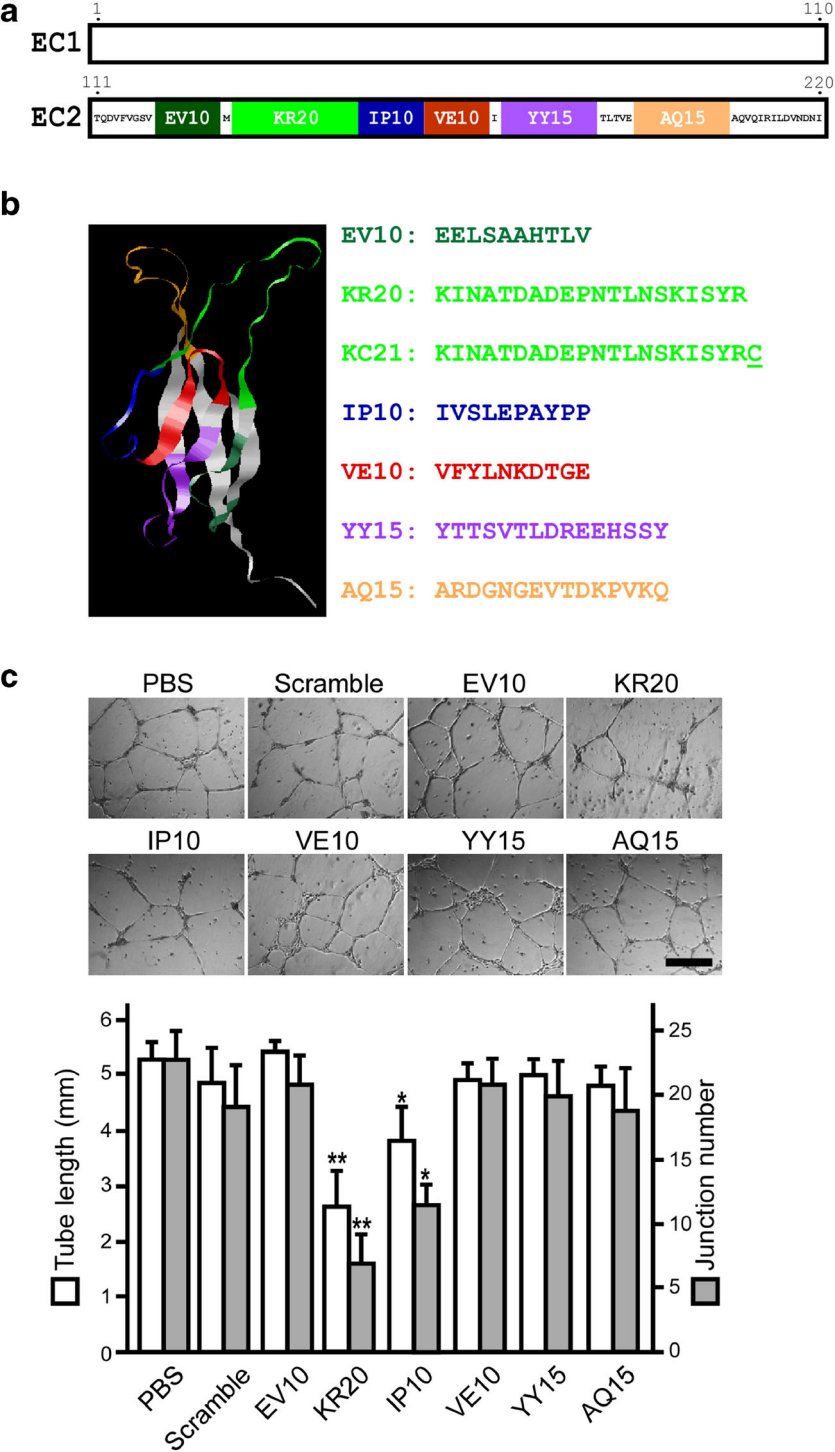
Design of Dsg2-derived peptides and test of their effects on ECFC tube-like structure formation **a** Scheme presentation of the EC1 (open box) and EC2 domains of Dsg2 protein. The six peptide fragments for test in this study are named and colored as indicated. The sequences of EC2 not covered by the chosen six peptides are shown by single letter. Numbers indicate the amino acid residues at the end of EC1 and EC2. **b** SWISS-MODEL protein structure homology-modeling server simulates 3D structure of EC2 domain. The six peptide fragments located in the corresponding 3D structure are colored. Each peptide sequence is shown. KC21 is derived from KR20 with one cysteine modification at C-terminal. **c** Representative images of ECFCs treated with Dsg2-derived peptides (all in 400 μM) show various effects on tube formation after 16 h of culture. In lower panel, total tube length (open box) and junction number (filled box) per field were measured by Image-Pro Plus 6.0 (Rockville, MD). Values are mean ± SD of triplicate assays from 3 independent experiments. *p < 0.05, **p < 0.001, compared with PBS treated cells (as control). Scale bar, 300 μm.

Dsg2 is an integral membrane protein with five tandem cadherin domains (EC1-EC5) [22]. The apposed EC1 domains form a strand swap dimer connecting adjacent cells [23, 24]. We synthesized six peptide fragments corresponding to EC2 domain which is not involved in Dsg2 homotypic interaction [23, 25] (Fig. 1a). As shown by Matrigel tube formation assay, KR20 is the most potent peptide in inhibiting human ECFC angiogenic potential with regard to the decrease of average tube length and junction number (Fig. 1c). The KR20 peptide was modified with a cysteine residue at C-terminus which is required for carrier protein coupling and exactly the same sequence for Dsg2 antibody generation previously [15]. We named the epitope sequence as KC21 (Fig. 1b). Both KC21 and Dsg2 antibody profoundly inhibit ECFC tube formation (Fig. S2). The comparison of KC21 with its parental peptide KR20 on suppressing ECFC tube formation shows similar effects, suggesting one cysteine residue modification does not affect the antiangiogenic activity (Fig. S3).

### KC21 Peptides Specifically Inhibit ECFC Tube Formation but Not Viability and Proliferation

**Fig. 2:**
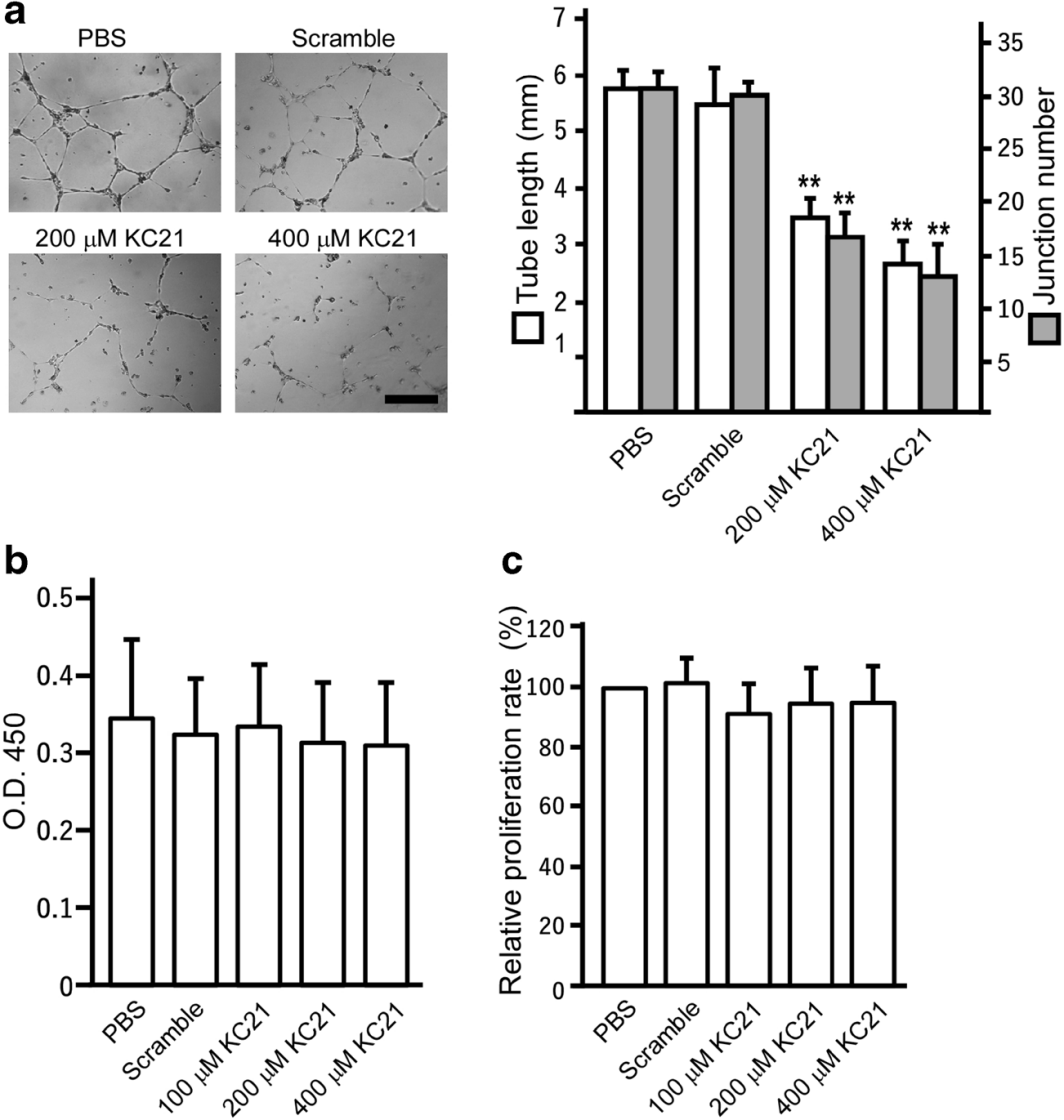
KC21 peptides inhibit ECFC tube formation but not viability and proliferation In **a**, representative images of ECFC tube-like structure in Matrigel culture. ECFCs were treated with indicated peptides for 16 hours. Right, quantification of total ECFC tube length and junction number per field. **p < 0.001, compared with PBS treated cells. Scale bar, 300 μm. **b** ECFCs were cultured with a series of KC21 or scramble peptides (400 μM) for 16 h and viability was measured by CC8 kit. **c** ECFCs were treated with a series of KC21 or scramble peptides (400 μM) for 24 h and proliferation rate was compared by BrdU incorporation assay.

At first, we measured the effects of KC21 peptides on inhibiting ECFC angiogenic potential, showing in a dose-dependent manner. KC21 is with similar potency to its parental peptide KR20 (Fig. 2a and S3). Effects of the peptides on ECFC tube formation are bio-comparable, as they do not change ECFC viability and proliferation rate determined by cell counting kit-8 (CCK8) assay and BrdU incorporation assay, respectively (Fig. 2b, c).

### KC21 Peptides Do Not Co-localize with and Decrease the Level of Dsg2

**Fig. 3:**
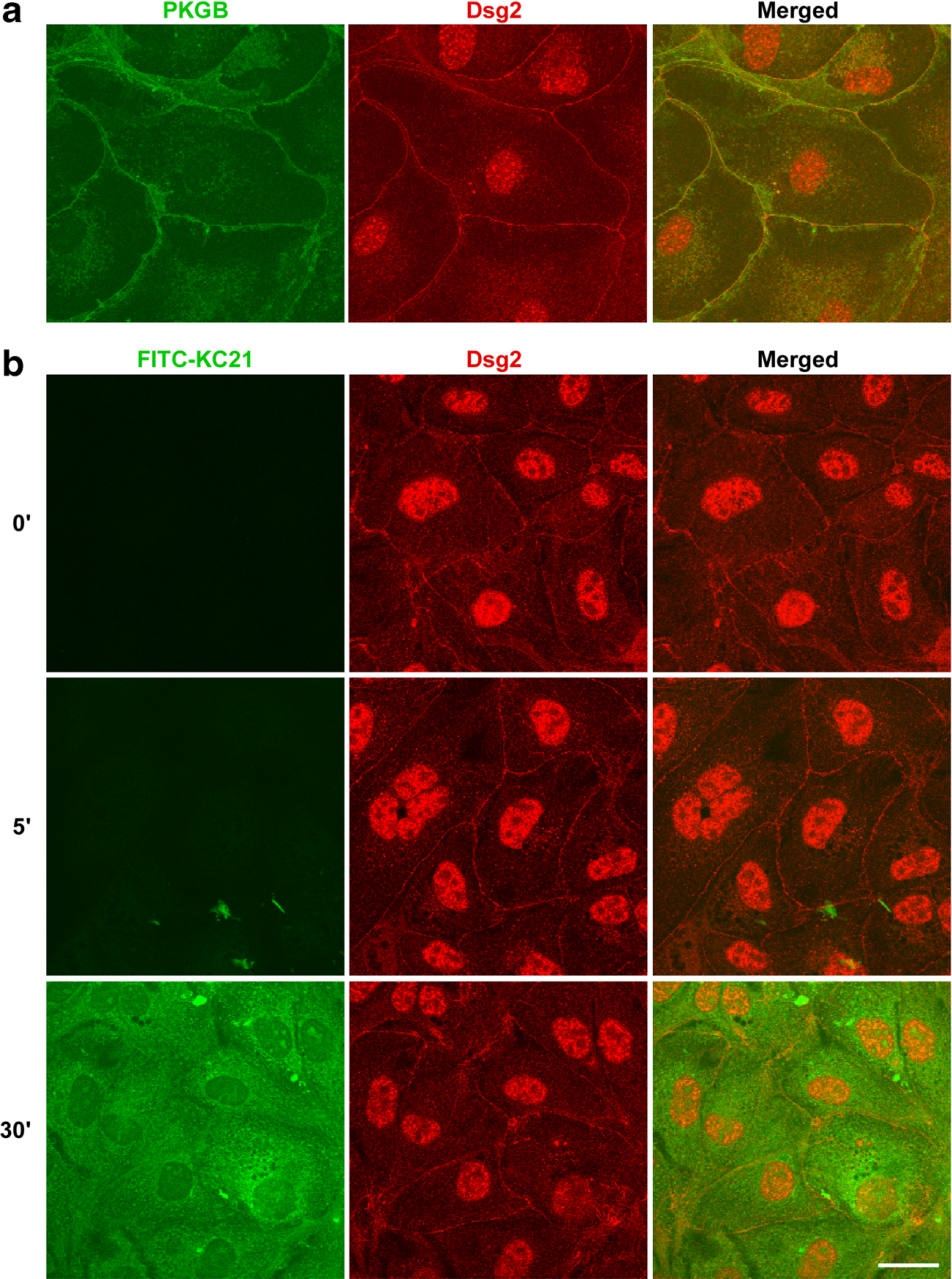
KC21 peptides do not interact with Dsg2 and do not affect the expression pattern of Dsg2 **a** Desmosomal protein plakoglobin (PKGB, green) was co-localized with Dsg2 (red) at the cell periphery of MDCK cells. **b** MDCK cells were treated with 400 μM of FITC-KC21 for the indicated time. Cells were fixed and stained with anti-Dsg2 antibodies. Images were acquired by confocal microscope. Scale bar, 25 μm.

We used Madin-Darby Canine Kidney (MDCK) cells, an epithelial cell line well known for the expression of functional desmosomes, to detect Dsg2 expression pattern. As shown in Fig. 3a, Dsg2 was co-localized with plakoglobin (PKGB), a Dsg2-associated protein at the periphery with a clear cell–cell boundary. The expression of Dsg2 in MDCK cells and ECFCs were also examined by flow cytometry (Fig. S4).

To test whether KC21 interacts with Dsg2 in vivo, the N-terminus of KC21 was conjugated with FITC (indicated as FITC-KC21, green) to track its cellular distribution. FITC-KC21 was taken up by MDCK cells within 30 min after treatment (Fig. 3b, left column). Dsg2 strongly decorated cell periphery (Fig. 3b, red), however, there was no co-localization of Dsg2 with KC21. Of note, the level of Dsg2 was not affected by KC21 treatment, as shown in western blot assay, the protein levels of Dsg2 were not changed in ECFCs treated with different concentrations of KC21 (Fig. S5).

### KC21 Peptides Inhibit ECFC Migration and VEGF-Induced p38 Kinase Activation

**Fig. 4:**
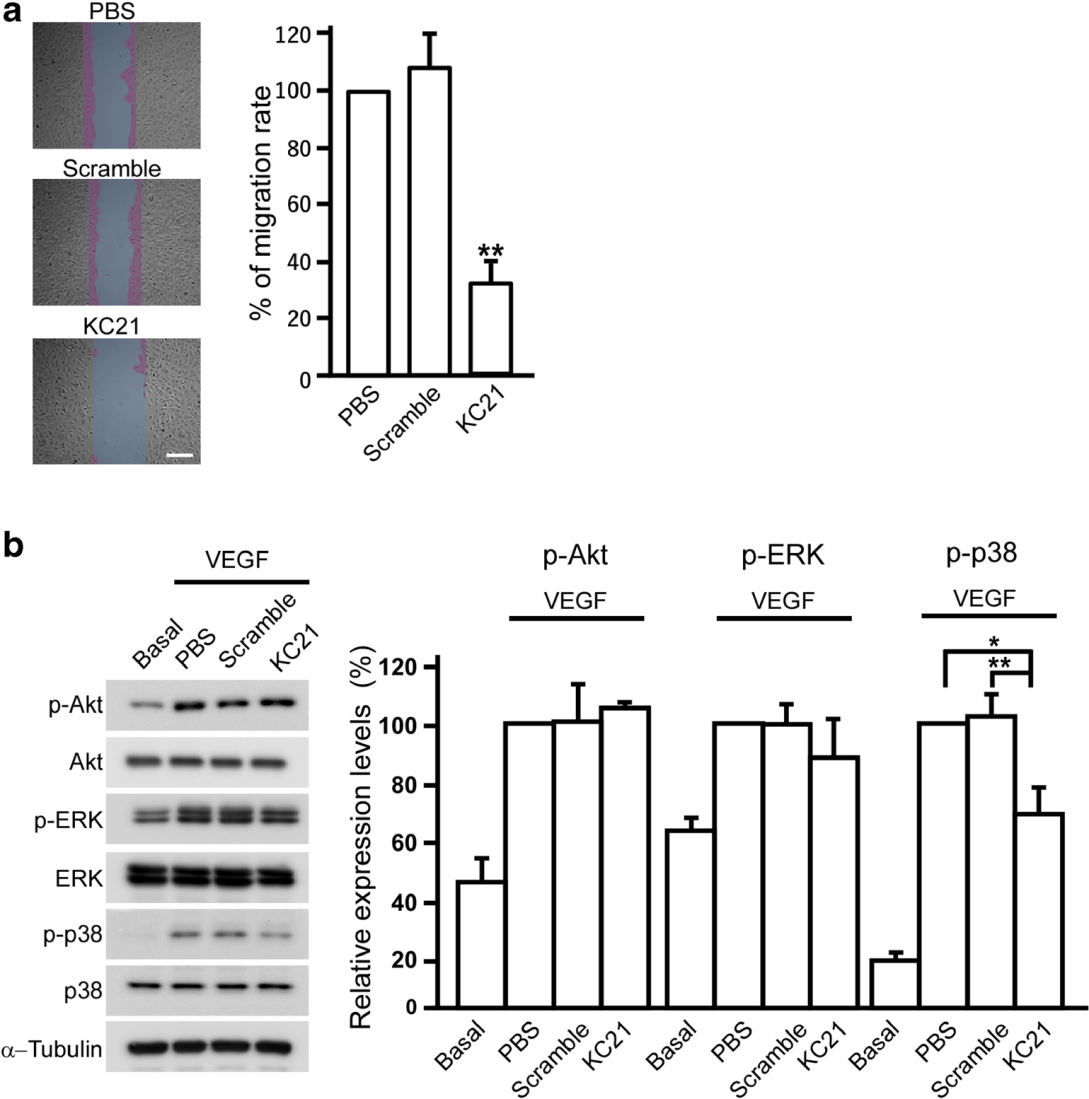
KC21 peptides inhibit ECFC migration and VEGF-induced p38 kinase activation **a** Representative images (left) and quantification results of wound healing assay. Gaps were formed (cyan) after inserted plugs removed from confluent ECFCs. The cells were then treated with scramble or KC21 peptides (400 μM for each) for 4 h. New growth areas after 4-hour culture were colored magenta and quantified. **p < 0.001. **b** Western blot of protein kinases induced by VEGF (10 ng/ml) in ECFCs treated with scramble or KC21 peptides (400 μM) for 24 hours and quantification results (right). The intensity ratio of phosphorylated kinase versus non-phosphorylated kinase was normalized with the intensity of loading control, α-Tubulin. Each group was compared with PBS group which was set as 100%. Values are mean ± SD of triplicate assays from 3 independent experiments. *p < 0.05; **p < 0.001, compared with PBS-treated cells.

As KC21 peptides show an antiangiogenic potential, we next asked whether KC21 inhibits ECFC migration, the prerequisite step of angiogenesis. As shown in Fig. 4a, the migrating activity of ECFCs is decreased after KC21 peptides treatment in wound healing assay.

VEGF is a major angiogenic factor in regulating PI3K/Akt for endothelial cell survival, PLC-γ/ERK for proliferation and p38 MAPK for cell migration [26–29]. KC21 peptides did not attenuate VEGF-induced Akt activity and ERK activation (Fig. 4b). Consistent with the moderate inhibition effects on ECFC migration, KC21 peptides cause a decrease of p38 MAPK phosphorylation triggered by VEGF.

### KC21 Peptides Inhibit VEGF-Induced Capillary Growth and Decrease MMP9 Activity

**Fig. 5:**
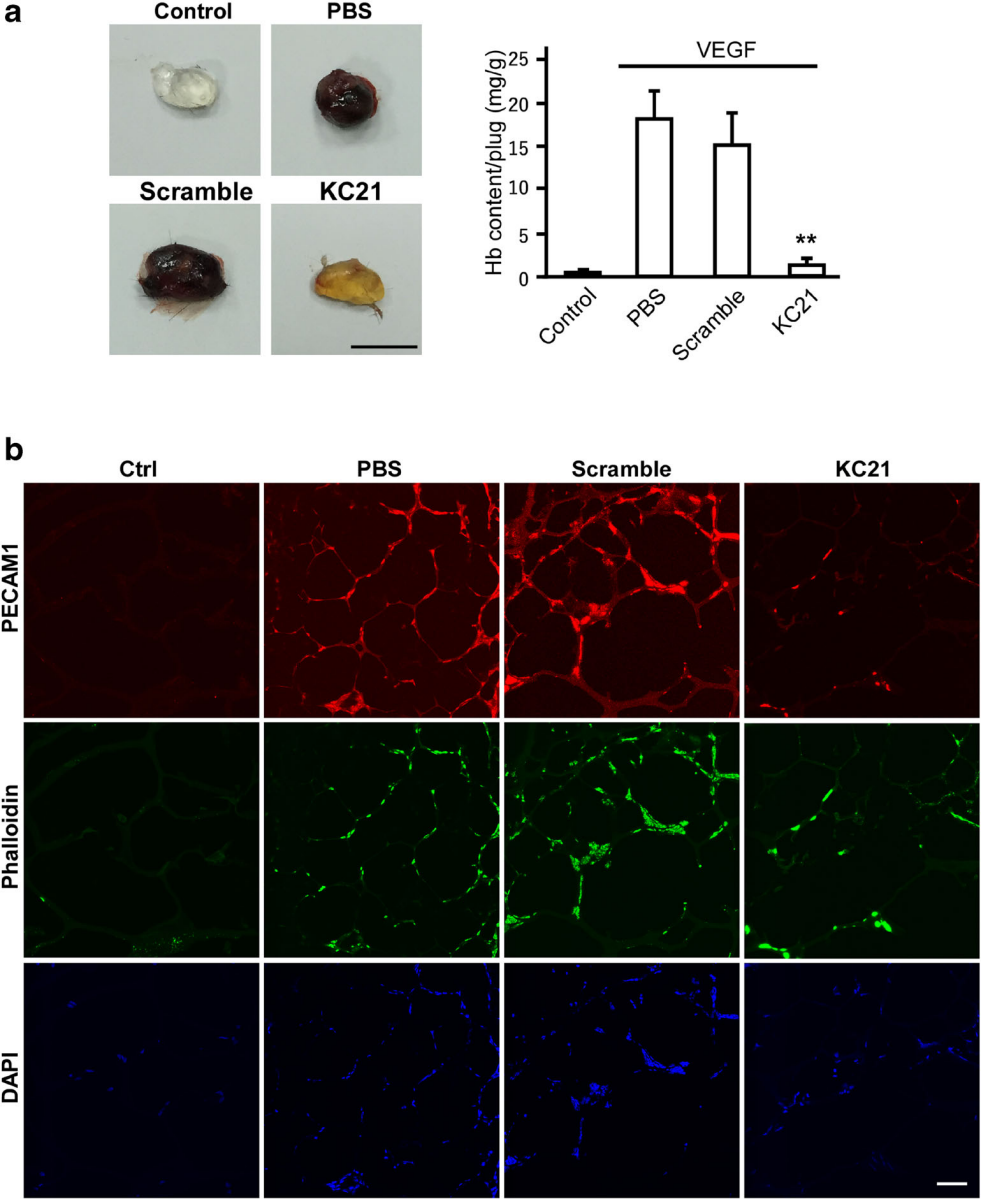

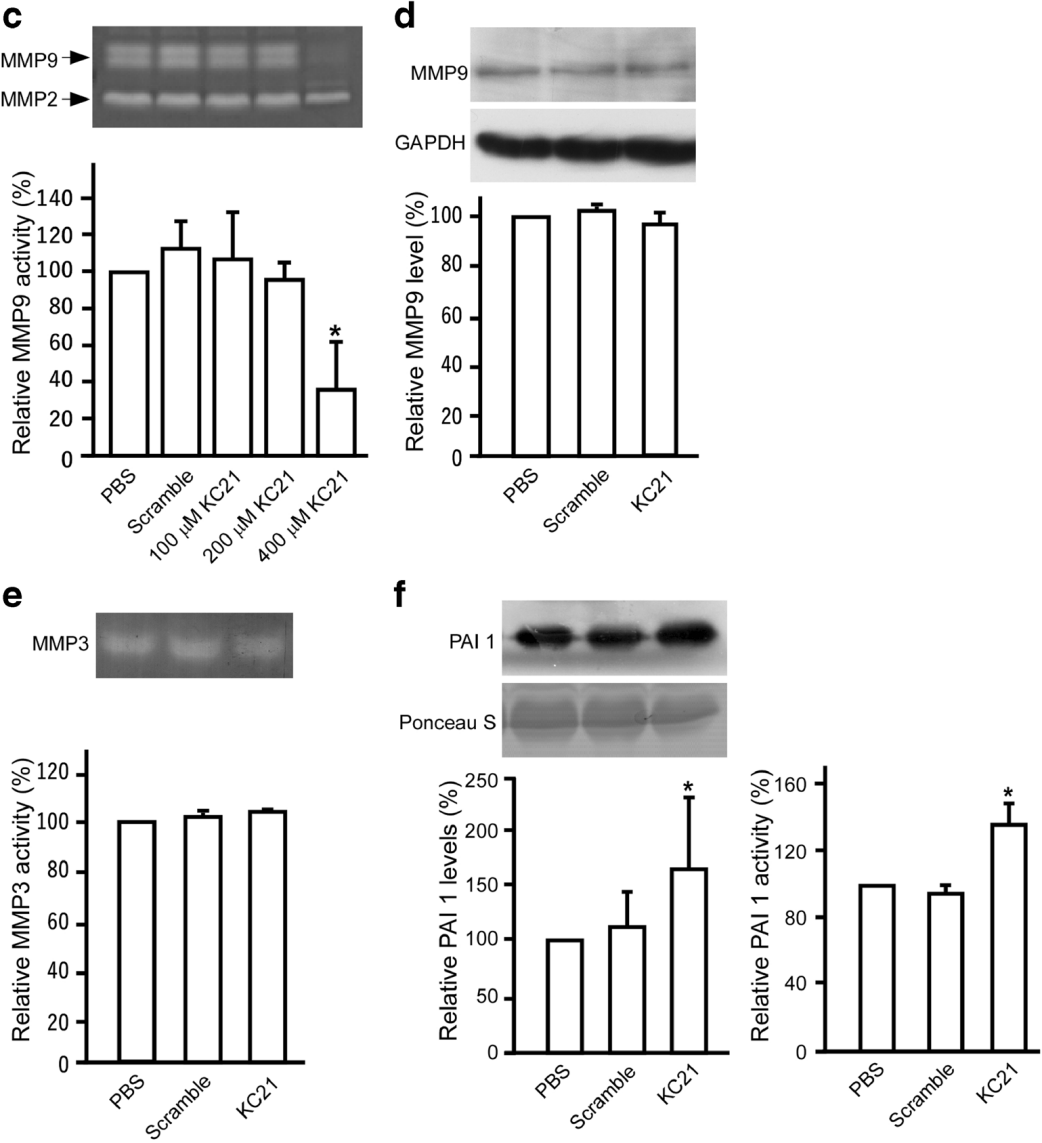
KC21 peptides inhibit VEGF-induced capillary growth and decrease MMP9 activity **a** Representative images of excised Matrigel plugs (left) and quantification of hemoglobin (Hb) content (right). Matrigel (0.5 mL) containing VEGF (10 ng/mL, with the exception of control) and KC21 peptides or scramble peptides (all in 400 μM) were subcutaneously injected into the mice and removed 7 days later. Matrigel with ingress of capillary tufts displays dark brown color. Hemoglobin was extracted and quantified. **p < 0.001, compared with PBS group. Scale bar, 10 mm. **b** Immunostaining of Matrigel plug sections (10 μm for each). Capillaries and infiltrated cells are stained with PECAM1, phalloidin, and DAPI (blue). **c** Gelatin zymography assay for MMP2 and MMP9 (upper) and quantification results of MMP9 activity (lower). **d** Western blot of MMP9 levels in harvested ECFCs and quantification results (lower). GAPDH is for a loading control. **e** Casein zymography assay for MMP3 activity (upper) and quantification results (lower). **f** Western blot of PAI-1 in conditioned media of ECFCs (upper) and quantification results (lower). Ponceau S staining is a loading control alternative to GAPDH as described [39]. Lower right, ECFC conditioned media were harvested and subjected to PAI-1 activity measurement. Values are mean ± SD of triplicate assays from 3 independent experiments. *p < 0.05 compared with PBS (untreated control) cells.

Since KC21 inhibits ECFC tube-like structure formation in vitro, we tested whether it inhibits VEGF-induced angiogenesis in vivo by subcutaneous Matrigel plugs assay. VEGF (10 ng/mL) profoundly induced capillary growth in plugs containing PBS and scramble peptides, while the induction was significantly abolished by KC21 peptides (Fig. 5a). The content of hemoglobin, an indicator of infiltrating erythrocytes, was markedly decreased in KC21-containing plugs (Fig. 5a, bar chart). The Matrigel plugs were sectioned and stained with CD31 (PECAM1) to detect capillaries and infiltrated cells (Fig. 5b). KC21 profoundly inhibits the angiogenic effects of VEGF.

Digestion of the extracellular matrix is the initial step of angiogenesis. Therefore, we further tested the effects of KC21 on angiogenic MMPs in ECFC culture. The results showed that KC21 peptides specifically inhibit the activities of extracellular MMP9, but not MMP2 or MMP3 (Fig. 5c, e). The intracellular MMP9 protein levels remain unchanged in ECFCs with KC21 treatment (Fig. 5d), suggesting KC21 regulates the activity instead of the amount of MMP9.

It has been proposed that activation of MMP9 is regulated by plasminogen/plasmin fibrinolytic system, controlled by plasminogen activator inhibitor-1 (PAI-1) [30]. We tested the effects of KC21 peptides in regulating the activity and cellular level of PAI-1. As shown in Fig. 5f, the level and activity of PAI-1were increased in ECFCs with KC21 peptide treatment.

### KC21 Peptides Suppress ECFC Angiogenesis and Inhibit Hyperoxia-Induced Retinal Neovascularization

**Fig. 6:**
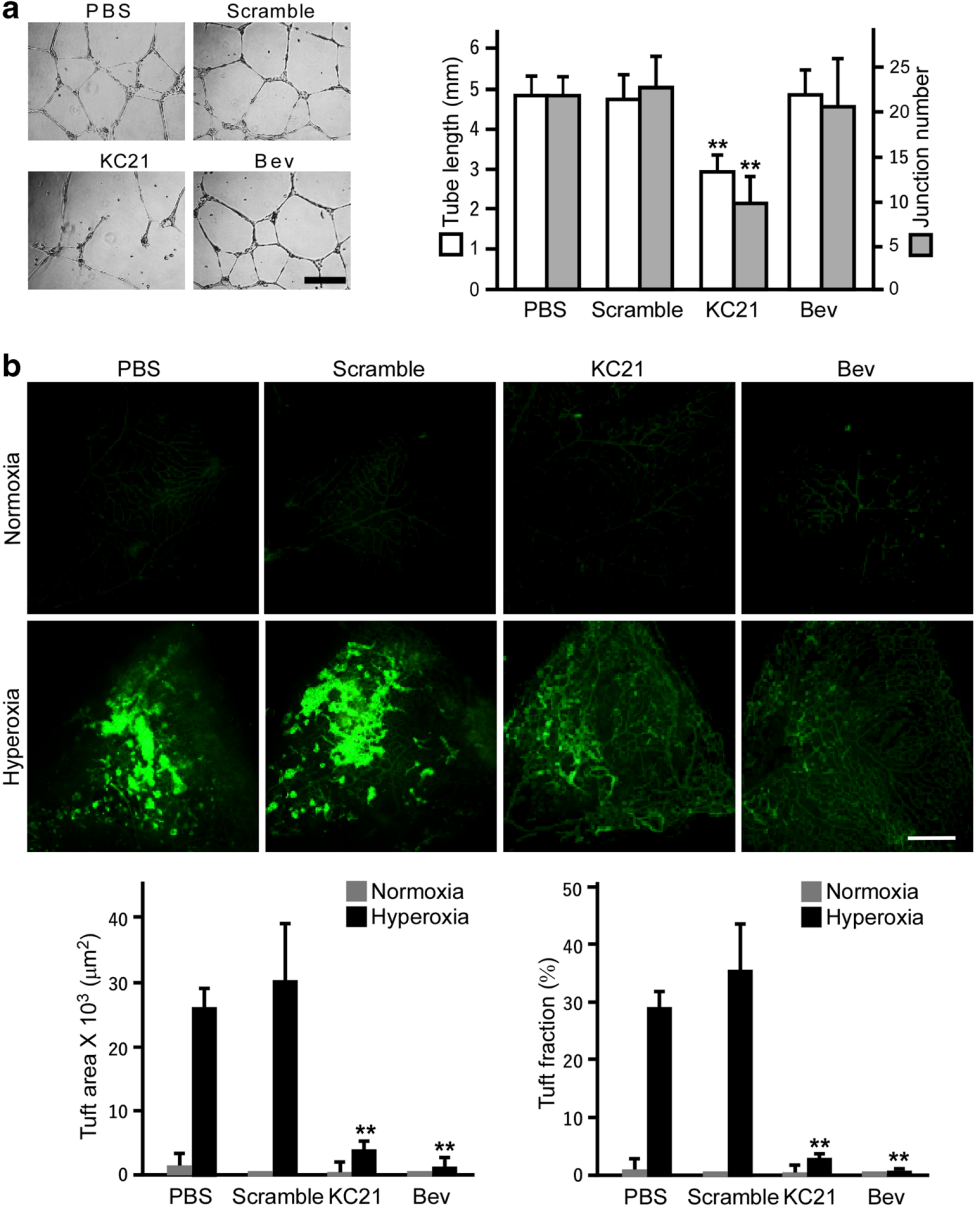
KC21 peptides suppress ECFC tube formation and inhibit hyperoxia-induced retinal neovascularization. **a** Representative images of tube formation assay of ECFCs treated with KC21 (400 μM) or bevacizumab (Bev, 20 ng) overnight and quantification results (right). **b** Images of retina obtained from mouse OIR model and quantification results of hyperoxia group (lower). Retina vessels were visualized by fluorescein-labeled isolectin-B4 staining. Tuft areas were measured using Image-Pro Plus 6.0 software (Rockville, MD). Values are mean ± SD from three independent experiments. **p < 0.001, compared with PBS group. Experiments were repeated for at least three times. Scale bars, 300 μm in A and 100 μm in B

In order to evaluate the antiangiogenic effect of KC21 on pathogenic neovascularization, we compared the treatments of KC21 with Bev, an FDA-approved therapeutic antibody for AMD. As shown in Fig. 6a, KC21 peptides inhibit ECFC tube formation, while bev has no effects. Furthermore, we used a well-established mouse oxygen-induced retinopathy (OIR) model to test the therapeutic effects of KC21 peptides on neovascularization. In normoxia condition, limited antiangiogenic effects are observed in mice with either KC21 (400 μM in 1 μl volume) or Bev (20 ng in 1 μl volume) treatment (Fig. 6b, upper 4 panels), while in hyperoxia condition, the induction of neovascularization is blunt by bev intravitreal injection (Fig. 6b, lower 4 panels). KC21 injection markedly inhibited retina neovascularization, indicated by the intensity of endothelial cells stained with isolectin-B4.

## Discussion

Our study showed that the designed KC21 peptides have the ability to inhibit human ECFC migration in vitro, to reduce VEGF-induced capillary growth and to suppress oxygen-induced retinal neovascularization in vivo. At the cellular level, KC21 peptides specifically attenuate VEGF-induced activation of p38 MAPK, but not the signaling targets of Akt and ERK. Also, KC21 peptides regulate MMP9 and PAI-1 to stabilize the extracellular matrix of ECFCs.

The functions of Dsg2 on angiogenesis have gained attention recently. Depletion of Dsg2 by siRNA impaired tube-like structure formation in MVECs [31]. These findings suggested that Dsg2 is a molecular target in regulating angiogenesis. Consistently, Dsg2 antibody profoundly inhibits tube-like structure formation (Fig. S2). The effects of Dsg2 antibody and KC21 on ECFC angiogenesis inhibition are comparable to the application of Dsg2 antibody and EC2 domain peptides on disturbing intercellular barriers in human colon carcinoma (Caco) enterocytes [32]. Our previous study also showed that Dsg2 antibody and the immunogenic epitope derived from EC2 domain suppress EMT and metastasis of human cancer cell lines [15]. As EC2 domain is not involved in Dsg2 homotypic interaction and Dsg2 is ubiquitously distributed in ECFCs, the effects of KC21 and Dsg2 antibody on suppressing ECFC angiogenesis and migration support the observation that Dsg2 exhibits a non-adhesive function to regulate cell migration and tissue morphogenesis [1, 5, 6]. Our results suggest that KC21 peptides may attenuate ECFC migration through inhibiting VEGF-mediated p38 activation (Fig. 4), consistent with the finding that p38 MAPK mediates VEGF-induced migration in HUVECs [33].

Proteolysis of extracellular matrix is an initiation step for the recruitment of endothelial progenitor cells to establish new capillaries. Matrix metalloproteinases (MMPs) are extracellular endopeptidases selectively degrading components of the extracellular matrix. Shedding Dsg2 ectodomains by MMPs had been detected in the inflamed intestinal mucosa of mice with colitis and patients with ulcerative colitis [7]. In this study, KC21 peptides specifically inhibit the activity of MMP9 but not MMP2 and MMP3 (Fig. 5c, d), consistent with the results that ectodomains of Dsg2 are substrates of MMP 9 but not of the other MMPs [7]. As MMP9 activity is regulated by plasmin and inhibited by PAI-1, increase of PAI-1 cellular level and activity by KC21 peptides may further inhibit MMP9 activity (Fig. 5e).

The therapeutic functions of mature endothelial cells on ischemic diseases have been tested in hindlimb ischemic animal model [34]. Both KC21 and bev strongly suppress angiogenesis in mature endothelial cells including human aortic endothelial cells (HAECs) and human umbilical vein endothelial cells (HUVECs) (Fig. S6). However, to our surprise, KC21 profoundly inhibits ECFC tube formation (Fig. 6a), while bev has no such effect. As both mature ECs and ECFCs contribute to the progression of pathogenic angiogenesis [35–37], KC21 might be a more potent peptide drug than bev on ocular neovascularization control.

To our knowledge, this study is the first report showing evidences for Dsg2-derived peptides to suppress retinal neovascularization in the mouse oxygen-induced-retinopathy (OIR) model. Neovascularization causes ocular vessel leakiness, edema, retinal detachment, and even blindness. VEGF is a major hypoxia-induced angiogenic factor and is found to be increased in the vitreous and retina to exacerbate retinopathy. Clinically, therapeutic agents against VEGF, such as bev and aflibercept, have been widely used to control the overgrowth of retinal blood vessels for vision rescue. However, more than 30% of the patients do not respond to these therapies and adverse events were also reported [38]. The finding in this study that the inhibitory effects of KC21 peptides on neovascularization in the mouse OIR model is comparable to that of Bev (Fig.6b) provides a new potential target for developing alternative or combined therapeutic options for retinal vascular diseases.

## Electronic supplementary material

ESM 1 Phenotypic characterization of human ECFCs and effects of KC21 peptides on ECFC and endothelial cell angiogenesis (DOCX 1395 kb)

## Abbreviations

Dsg: desmoglein
Bev: bevacizumab
OIR: oxygen-induced retinopathy
AMD: age-related macular degeneration
VEGF: vascular endothelial growth factor
MMPs: matrix metalloproteinases
PAI-1: plasminogen activator inhibitor 1
EPCs: endothelial progenitor cells
ECFCs: endothelial colony forming cells
MVECs: microvascular endothelial cells
ECs: endothelial cells
CCK-8: the cell counting kit 8
PBS: phosphate buffer saline
