# Supplementary material for: KC21 Peptide Inhibits Angiogenesis and Attenuates Hypoxia-Induced Retinopathy
Source: J Cardiovasc Transl Res. 2019 Feb 21;12(4):366–77. doi: 10.1007/s12265-019-09865-6 (PMC6707963; doi:10.1007/s12265-019-09865-6)
Supplement: Supplementary file 1 — Phenotypic characterization of human ECFCs and effects of KC21 peptides on ECFC and endothelial cell angiogenesis (DOCX 1395 kb) [file 12265_2019_9865_MOESM1_ESM.docx]

**Phenotypic characterization of human ECFCs and effects of KC21 peptides on ECFC and endothelial cell angiogenesis**

Chi-Sheng Lu ^1*^, Yi-Nan Lee^1*^, Shin-Wei Wang ^2^, Yih-Jer Wu^1,2^, Cheng-Huang Su^1^, Chin-Ling Hsieh^1^, Ting Yi Tien^1^, Bo Jeng Wang^2^, Min-Che Chen^3^, Chun-Wei Chen^3^, Hung-I Yeh^1,2,⁋^

^1^Departments of Medical Research and Internal Medicine, Mackay Memorial Hospital, Taipei 10449, Taiwan

^2^Department of Medicine, Mackay Medical College, New Taipei City 25245, Taiwan

^3^Asclepiumm Taiwan Co., Ltd., New Taipei City 25160, Taiwan

^⁋^ Correspondence to: Hung-I Yeh ([hiyeh@mmh.org.tw](mailto:hiyeh@ms1.mmh.org.tw))

**Additional file**


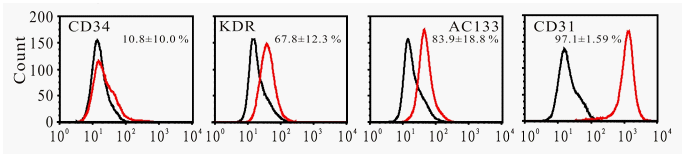


Fig. S1 Phenotypic characterization of ECFCs was performed after 28 days of culture.

Representative images of expression of endothelial markers CD34, KDR (VEGFR2), AC133 (CD133), and CD31 by flow cytometry. Black histograms are isotype antibodies staining and red histograms represent cells positively stained with CD34, KDR, AC133, or CD31. The percentage of ECFC expressing each marker is presented as mean ± SD (n = 6). Note that the number of PBMCs from 50 mL blood of healthy donors is about 1*10^4^ cells, and that of ECFCs used in the present study is about 6.4*10^7^ at passage 18.


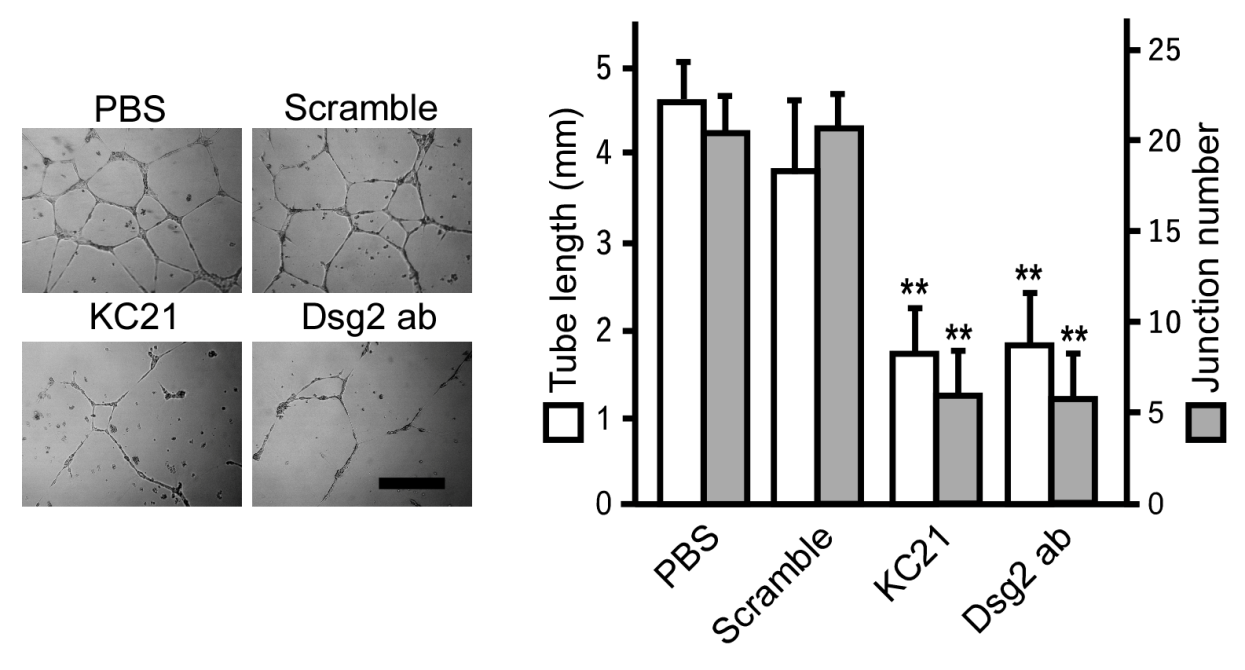


Fig. S2 Effects of KC21 and anti-Dsg2 antibody on ECFC tube formation.

Left, representative images of ECFC tube-like structure in Matrigel culture. ECFCs were treated with KC21 peptides or Dsg2 antibodies (ab) for 16 hours. Right, quantification of ECFC tube length and junction number per field. **, p< 0.001, compared with PBS treated cells. Scale bar, 300 μm.


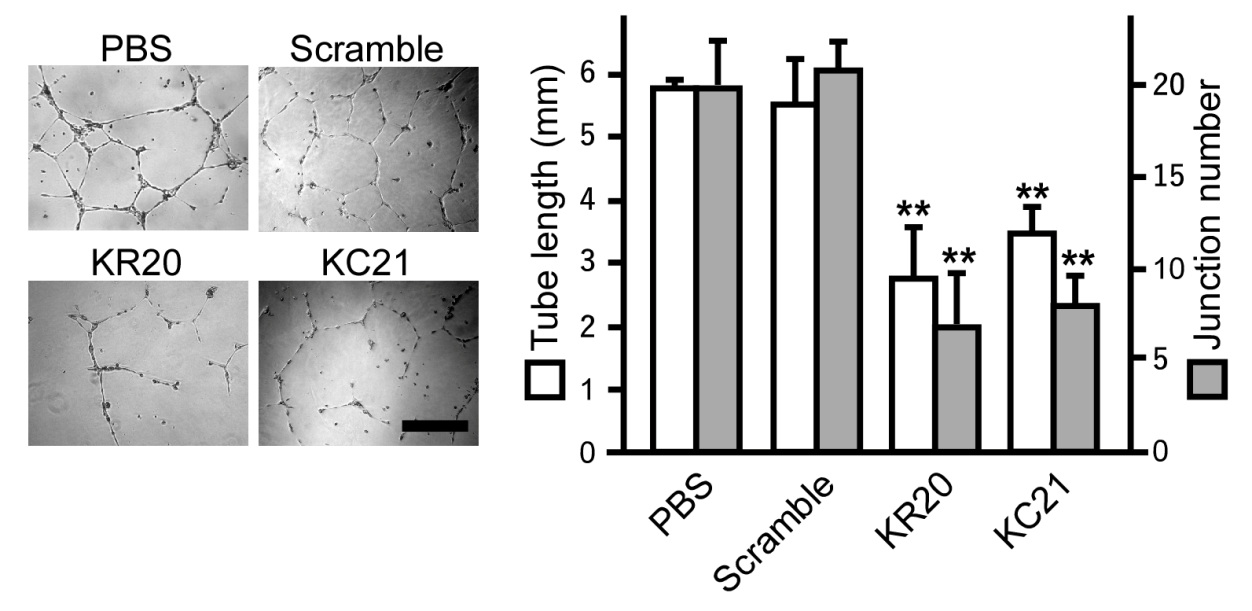


Fig. S3 Effects of KR20 and KC21 on ECFC angiogenesis.

Left, representative images of ECFC tube-like structure. ECFCs were treated with indicated peptides (all in 400 μM) for 16 hours. Right, quantification of ECFC tube length and junction number per field. **, p< 0.001, compared with PBS treated cells. Scale bar, 300 μm.


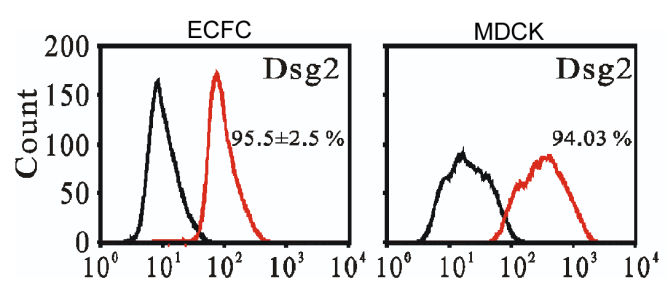


Fig. S4 Representative images of Dsg2 expression in ECFCs and MDCK cells assayed by flow cytometry. Black histograms are isotype antibodies staining and red histograms represent cells positively stained with Dsg2 antibodies. The percentage of ECFCs expressing each marker is presented as mean ± SD (n = 3). For MDCK, n = 1.


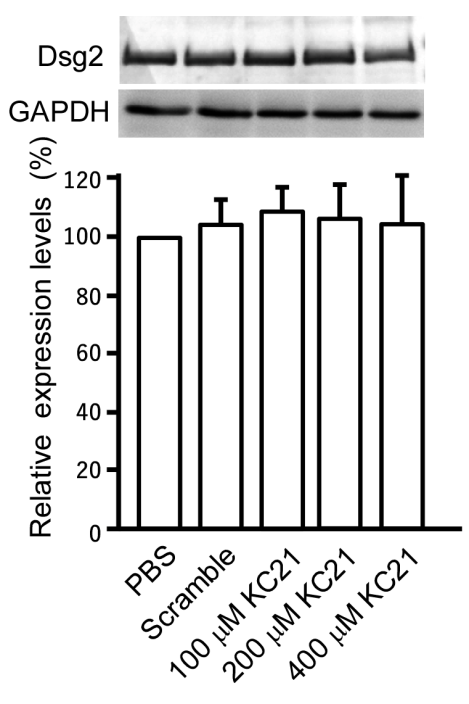


Fig. S5 KC21 peptides do not decrease Dsg2 level of ECFCs.

ECFCs were treated with scramble (400 μM) or KC21 (as indicated) overnight. Cell lysates were collected for Western blotting followed by quantification (n=3).


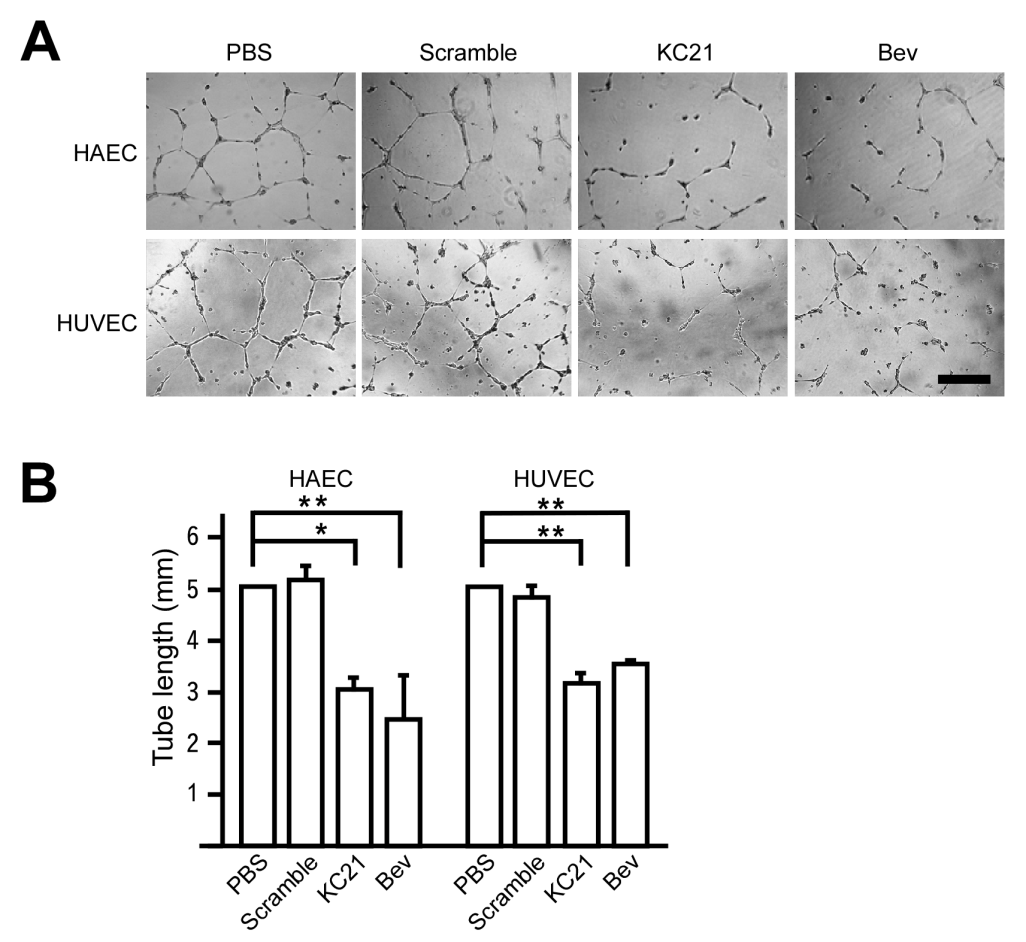


Fig. S6 Effects of KC21 and Bev on endothelial cell angiogenesis.

(A) Representative images of tube formation assay of HAECs and HUVECs treated with KC21 (400 μM) or Bev (20 ng). (B) Quantification results of average tube length per field. Experiments were repeated for at least three times. *, p < 0.05, **, p < 0.01, compared with PBS treated cells. Scale bar, 300 μm.
